# Bone regeneration in minipigs by intrafibrillarly-mineralized collagen loaded with autologous periodontal ligament stem cells

**DOI:** 10.1038/s41598-017-11155-7

**Published:** 2017-09-05

**Authors:** Ci Zhang, Boxi Yan, Zhen Cui, Shengjie Cui, Ting Zhang, Xuedong Wang, Dawei Liu, Ruli Yang, Nan Jiang, Yanheng Zhou, Yan Liu

**Affiliations:** 1 Laboratory of Biomimetic Nanomaterials, Department of Orthodontics, Peking University School and Hospital of Stomatology, National Engineering Laboratory for Digital and Material Technology of Stomatology, Beijing Key Laboratory of Digital Stomatology, Beijing, 100081 China; 2 Department of Stomatology, Beijing Tongren Hospital, Capital Medical University, Beijing, 100730 China

## Abstract

Biomimetic intrafibrillarly-mineralized collagen (IMC) is a promising scaffold for bone regeneration because of its structural and functional similarity to natural bone. The objective of this study was to evaluate the bone regeneration potential of IMC loaded with autologous periodontal ligament stem cells (PDLSCs) in large bone defects in minipigs. A macroporous IMC with a bone-like subfibrillar nanostructure was fabricated using a biomimetic bottom-up approach. Non-healing full thickness defects were established on the cranial bone in minipigs, and IMC and hydroxyapatite (HA) scaffolds seeded with autologous PDLSCs were implanted into these defects. Computed tomographic imaging, histology staining, and atomic force microscopy were applied to evaluate to the quantity, micro/nano structures, and mechanical performance of the neo-bone after 12 weeks of implantation. Compared with HA, IMC showed superior regeneration properties characterized by the profuse deposition of new bony structures with a normal architecture and vascularization. Immunohistochemistry showed that the runt-related transcription factor 2 and transcription factor Osterix were highly expressed in the neo-bone formed by IMC. Furthermore, the nanostructure and nanomechanics of the neo-bone formed by IMC were similar to that of natural bone. This study provides strong evidence for the future clinical applications of the IMC-based bone grafts.

## Introduction

The bone is a mineralized collagenous tissue with a complex hierarchical organization that remodels throughout one’s lifecycle to adapt to mechanical stress and maintain the integrity of skeletal tissues. The disequilibrium between bone resorption and bone formation in osteoporosis, large bone defects associated with severe trauma, surgical resections, and congenital malformations all require alternatives to reverse the process of bone loss and to regenerate bones^1^. Traditional bone repair approaches mainly focus on the use of bone grafts from autologous, allogeneic and xenogeneic sources. However, several complications such as donor-site morbidity, host immune rejection, and disease transmission, limit the application of these bone grafts^2–4^ and motivate the development of synthetic biomaterials, including ceramics, polymers, metals, and composites^5–7^.

Calcium phosphate bioceramics, such as hydroxyapatite (HA) and tricalcium phosphate, have been widely used as temporary scaffolds for bone regeneration because of their biocompatibility, osteoconductivity and chemical similarity to the mineral phase of human bones^8, 9^. However, their clinical applications have been limited because pure inorganic bioceramics lack the flexibility of organic polymers, the hierarchical microstructures of bones and a proper degradation rate^9, 10^. The new bone generated by HA as a scaffold took the form of a porous HA network, that cannot sustain the mechanical load for remodeling^11^. Therefore, microstructured inorganicorganic composites that consist of HA and biodegradable polymers, such as collagen, gelatin, chitosan, and poly(lactic-co-glycolic) acid, offer an alternative solution to some of the afore-mentioned drawbacks^12–15^. Given that natural bones are hierarchically-structured composites that comprise nanoscale hydroxyapatites that are periodically arranged within the gap zones of collagen fibrils^16–18^, mineralized collagen with a bone-like hierarchy is proven an ideal candidate for bone tissue engineering.

Multipotent mesenchymal stem cells (MSCs) are commonly used as seed cells for bone tissue engineering. Traditionally, bone marrow MSCs (BMSCs) are typical stem cells in bone regeneration. However, donor site morbidity limits the quantity of bone marrow for therapeutic use. Periodontal ligament stem cells (PDLSCs), which are part of a specific MSC population, can be easily isolated from extracted third molars^19, 20^. PDLSCs also show a greater capacity for bone and tendon regeneration than BMSCs^21, 22^.

Biomimetic bone-like intrafibrillarly-mineralized collagen (IMC) was successfully fabricated in our previous studies and was proven to be a biocompatible and osteoconductive material with a proper degradation rate^23–28^. An IMC combined with BMSCs could promote bone regeneration among rats with critical-sized mandibular bone defects^27, 28^. However, large animal studies with high clinical relevance are required to evaluate the bone regeneration potential of IMC^29, 30^. Given that porcine and human bones are highly similar in terms of their composition, microstructure, and remodeling process^31^, the purpose of this study was to investigate the potential of IMC loaded with autologous PDLSCs in regenerating the bones of large defects in minipigs and to provide highly relevant evidence to support the future clinical applications of IMC-based bone grafts.

## Materials and Methods

### Scaffold preparation

The 3-D macroporous IMC scaffold was prepared using a modified bottom-up biomimetic approach^27, 28^. Briefly, type I tropocollagen solution (Corning, 10 mg/mL, pH < 2) was slowly injected into a dialysis flask (3500 Da), which was immersed in a mineralization solution (pH = 7.4) containing poly(acrylic acid) (0.25 mM, Mw 2000, Sigma-Aldrich), type I white Portland cement (0.2 g/mL, Lehigh Cement Co.) and simulated body fluid. The reaction was performed at room temperature in a moisture chamber to minimize evaporation. The mineralization solution was changed every 3 days. After 7 days, the fibrillized collagen suspension was collected by centrifugation, poured into the plastic molds and lyophilized to form sponge-like porous scaffolds. To observe the microstructure of these scaffolds, the samples were dehydrated in a graded series of ethanol (50–100%), critical point dried, and examined via scanning electron microscopy (SEM, S-4800, Japan) at 15 kV. An elemental analysis of these scaffolds was performed using energy-dispersive X-ray spectroscopy (EDS) coupled with SEM. The cell-seeded scaffolds were washed three times with phosphate buffered saline (PBS) and fixed in 2.5% glutaraldehyde in PBS before SEM examination.

### Animals

The protocols in this section were approved by the Animal Use and Care Committee of Peking University (permit number: LA2014216). The methods employed were performed in accordance with approved guidelines. Nine male health-certified minipigs (22 months old, 54.1 kg mean weight, Guizhou Minipigs, China) were used in this study. The minipigs were housed individually and given free access to pig maintenance food and water. Before surgery, these animals were anesthetized with a combination of ketamine chloride (6 mg/kg) and xylazine (0.6 mg/kg).

### Isolation and identification of minipig PDLSCs

The minipig PDLSCs were isolated and cultured under the same conditions employed in our previous study for human PDLSCs^26^. Periodontal ligament tissue was scraped from the middle third of the root surface of the incisors in minipigs and was digested with collagenase type I (3 mg/mL; Worthington Biochemical, USA) and dispase II (neutral protease, 4 mg/mL; Roche Diagnostics, USA) for 2 h at 37 °C to obtain a single-cell suspension. After isolation, the cells were cultured in alpha minimum essential medium (Gibco, Thermo Fisher Scientific, Waltham, MA) with 20% fetal bovine serum (Hyclone; GE Healthcare Life Sciences, Logan, UT), 100 U/mL penicillin/streptomycin, 2 mM glutamine, 55 mM 2-ME (Gibco), and 0.1 mM L-ascorbic acid phosphate (Wako Chemicals, Richmond, VA) at 37 °C in 5% CO_2_. The 3rd passage of PDLSCs was used in the following experiments. The multipotency of the PDLSCs was confirmed by examining osteogenicity using Alizarin red S staining and adipogenicity using Oil red O staining (Fig. S1).

### In vitro cell seeding on different scaffolds

To examine the influence of scaffolds on cell growth and extracelluar matrix (ECM) secretion, minipig PDLSCs at passage 3 were seeded at 1 × 10^6^ cells/scaffold and were cultured with regular medium without any osteogenic supplements at 37 °C in 5% CO_2_. Before cell-seeding, the scaffolds were sterilized with 75% ethanol for 24 hours and were immersed in PBS with 100 U/mL penicillin/streptomycin overnight. On days 3 and 7, the scaffolds were fixed with 2.5% glutaraldehyde in PBS and lyophilized for SEM examination.

### Animal surgery

Two non-healing full thickness defects of approximately 2 cm width × 3 cm length × 0.5 cm depth were established on the cranial bone of each minipig and eighteen defects were randomly divided into experimental and control groups. The IMC (N = 6) and HA (N = 6) scaffolds (Dongbo Biotechnology, China) seeded with 1 × 10^6^ minipig PDLSCs were randomly placed into the defect area, and no implant was used in the negative control (N = 6). After 12 weeks of implantation, the minipigs were sacrificed via anesthesia overdose. The craniums were obtained from each group and fixed with 10% formalin in PBS after the removal of soft tissue.

### Computed tomographic (CT) measurements

To evaluate the formation of new bones by different scaffolds, the fixed craniums were scanned using CT imaging (Siemens Medical Solutions, Knoxville, TN, USA) at 80 kV and 500 mA. The Inveon Research Workplace software (Siemens, USA) was applied to calculate the ratio of bone volume/tissue volume (BV/TV) and bone mineral density (BMD) in the defect area. To calculate BV/TV, the gray value was set between 400 and 1200 to eliminate the influence of the residual scaffold on bone volume.

### Histological analyses

After CT scanning, half of the craniums were decalcified in 15% EDTA for 8 weeks, dehydrated in a graded series of ethanol (70–100%) and embedded in paraffin. Serial tissue sections with 5 μm thickness were prepared from the mid-sagittal plane of the defect area, treated with hematoxylin-eosin (HE) and Masson’s trichrome staining, and observed under a light microscope (Carl Zeiss Inc., Germany). The residual scaffold fraction was calculated as the residual scaffold area divided by the defect area using histomorphometric techniques. After Masson’s trichrome staining, the blue color indicates the regenerated bone, collagen fibers, or osteoid, while the red color indicates the mature bone.

### Immunohistochemical assessment

Immunohistochemistry was performed with a 2-step detection kit (Zhongshan Golden Bridge Biotechnology, Beijing, China). Deparaffinized sections were subjected to antigen retrieval by 0.125% trypsin and 20 μg/ml proteinase K solution. The sections were then blocked with 5% BSA and treated with the following antibodies: runt-related transcription factor 2 (Runx2; 1:300; orb10256, Biorbyt), rat zinc finger transcription factor Osterix (Osx; 1:300; PA5-40509, Thermofisher scientific), and transforming growth factor- Beta 1 (TGF-β; 1:200; LS-C10852, LSBio). After washing with PBS, the sections were subsequently incubated with horseradish peroxidase-conjugated secondary antibodies, using diaminobenzidine (Zhongshan Golden Bridge Biotechnology, Beijing, China) as chromogen.

### Atomic force microscope (AFM) measurements

The other half of the craniums were undecalcified and sectioned from the center of the defect areas. AFM (Dimension Icon, Bruker, USA) was applied to test the nanostructure and nanomechanics of the newly-formed bones under ambient conditions. Six regions of interest (ROIs) were selected, and the median value was used to represent the property value for each ROI. Thus, 18 values (3 samples × 6 ROIs) were generated for each group.

### Statistical analysis

All data were expressed as mean ± standard deviation and assessed via one-way ANOVA and Holm-Sidak pairwise comparison after performing normality and equal variance tests. Statistical significance was considered at P < 0.05.

All data generated or analysed during this study are included in this published article (and its Supplementary Information files).

## Results

### Characterization of scaffolds

**Figure 1:**
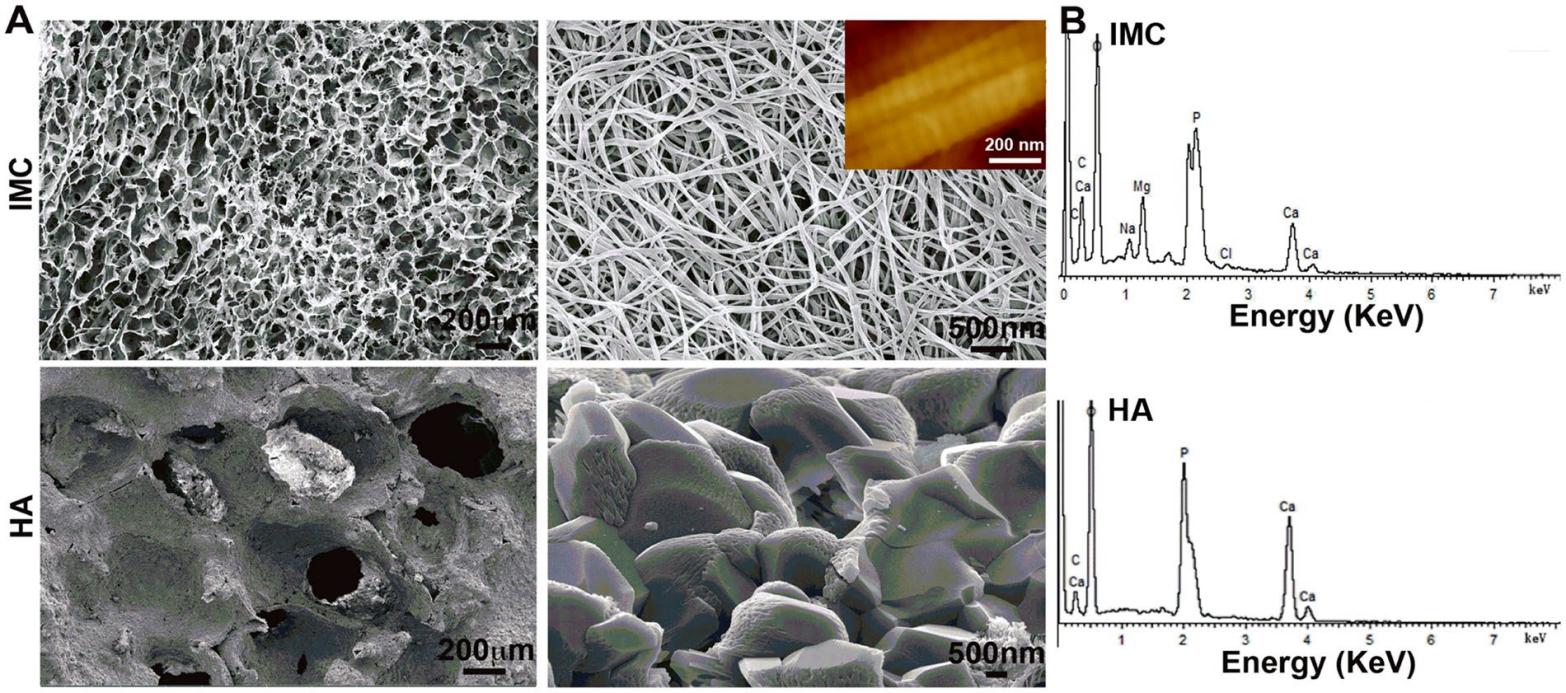
Morphological and elemental analysis of different scaffolds. (**A**) Representative (left) low- and (right) high-magnification SEM images of cross-sections of IMC and HA scaffolds. (**B**) EDS in different scaffolds, confirming the presence of apatite crystallites within the collagen fibrils in the IMC scaffold. Inset: AFM morphology of IMC showing obvious cross-banding patterns.

The IMC was fabricated using a modified biomimetic bottom-up approach, in which collagen fibrillogenesis and nanoapatite platelet formation occurred simultaneously. After free drying, 3-D porous IMC scaffolds were obtained and showed a spongy morphology with interconnected macropores of 148.2 ± 46.5 μm (Fig. 1). Under high magnification, the collagen fibrils exhibited bone-like subfibrillar nanostructure with cross-banding patterns (Inset). No apatite crystals were formed around the fibrils, suggesting that the mineral phase is primarily embedded within the fibrils. The presence of nanoapatites within the collagen fibrils was revealed by EDS, in which the Ca/P ratio was 1.52 ± 0.06, indicating of calcium-deficient nanoapatites. The HA scaffold composed of micro-sized apatites showed ununiform macropores of 345.7 ± 47.4 μm and the Ca/P ratio was 1.61 ± 0.03.

### Matrix secretion by PDLSCs on different scaffolds

**Figure 2:**
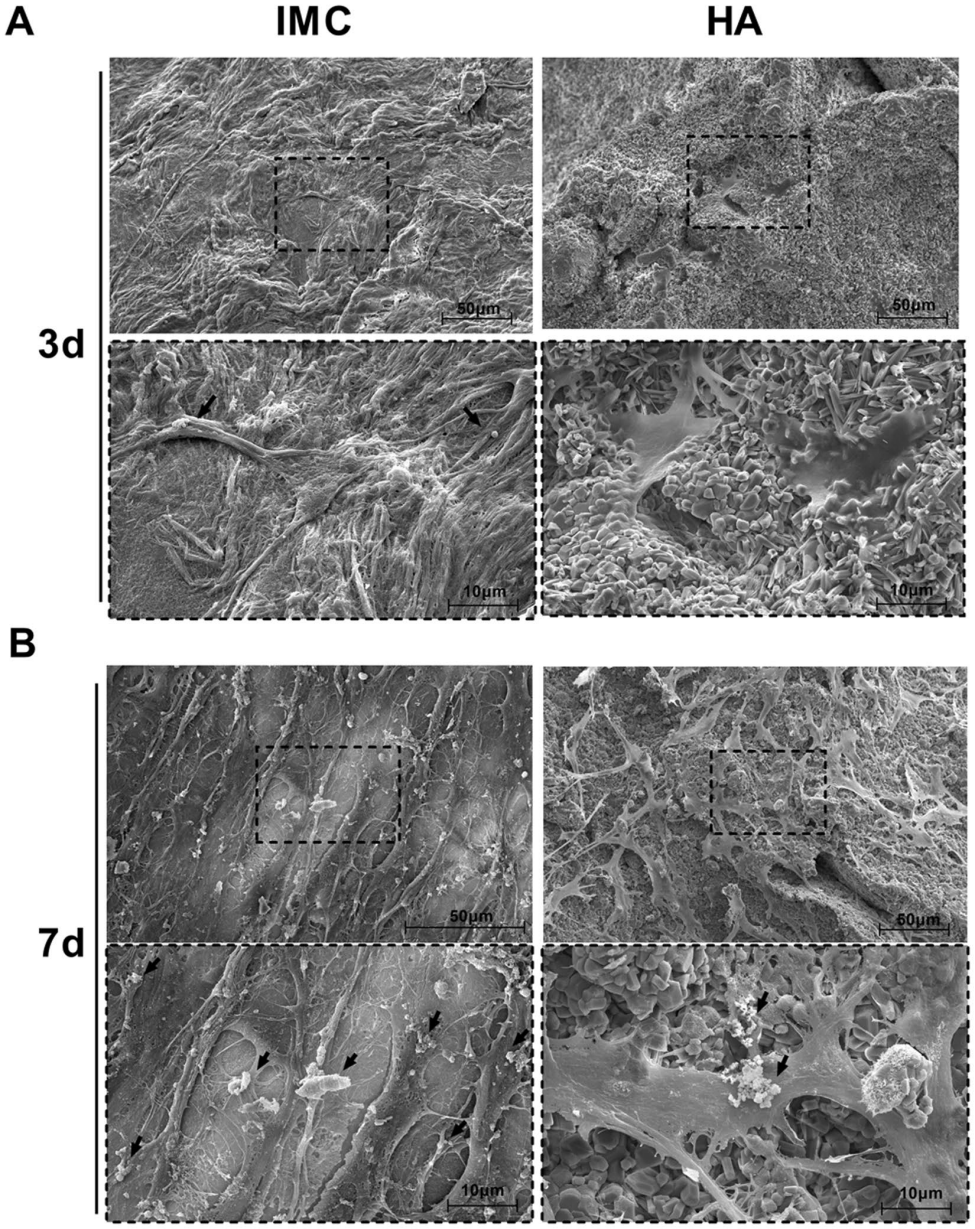
Representative SEM images of PDLSCs cultured on different scaffolds. (**A**) On day 3, PDLSCs attached to all the scaffolds, but only secreted ECM (black arrows) on the IMC scaffold. (**B**) On day 7, abundant fibrous ECM was secreted in the IMC group and calcified nodules deposited on the cell surface in the HA group.

The primary minipig PDLSCs showed typical fibroblastic morphology with a spindle shape and formed evident clones after three days of culture. The positive staining of Alizarin red and Oil red confirmed the osteogenic and adipogenic differentiation ability of PDLSCs (Fig. S1). To observe cell-scaffold interactions, the identified PDLSCs were seeded on different scaffolds. After 3 days of culture, PDLSCs contacted well with the IMC and HA scaffolds via their filopodia. The cells were spread all over the surface of the IMC scaffold and secreted some ECM, whereas few cells without ECM secretion were observed on the surface of the HA scaffold (Fig. 2A). After 7 days of culture, the PDLSCs seeded on the IMC developed cytoplasmic extensions with abundant cell-cell junctions, and produced numerous matrix vesicles that were attached to the cell membranes. By contrast, the PDSLCs seeded on HA scaffold showed a less extensive ECM secretion with large calcium nodules deposited on the cell surface (Fig. 2B).

### CT analysis of new bone volume in minipigs

**Figure 3:**
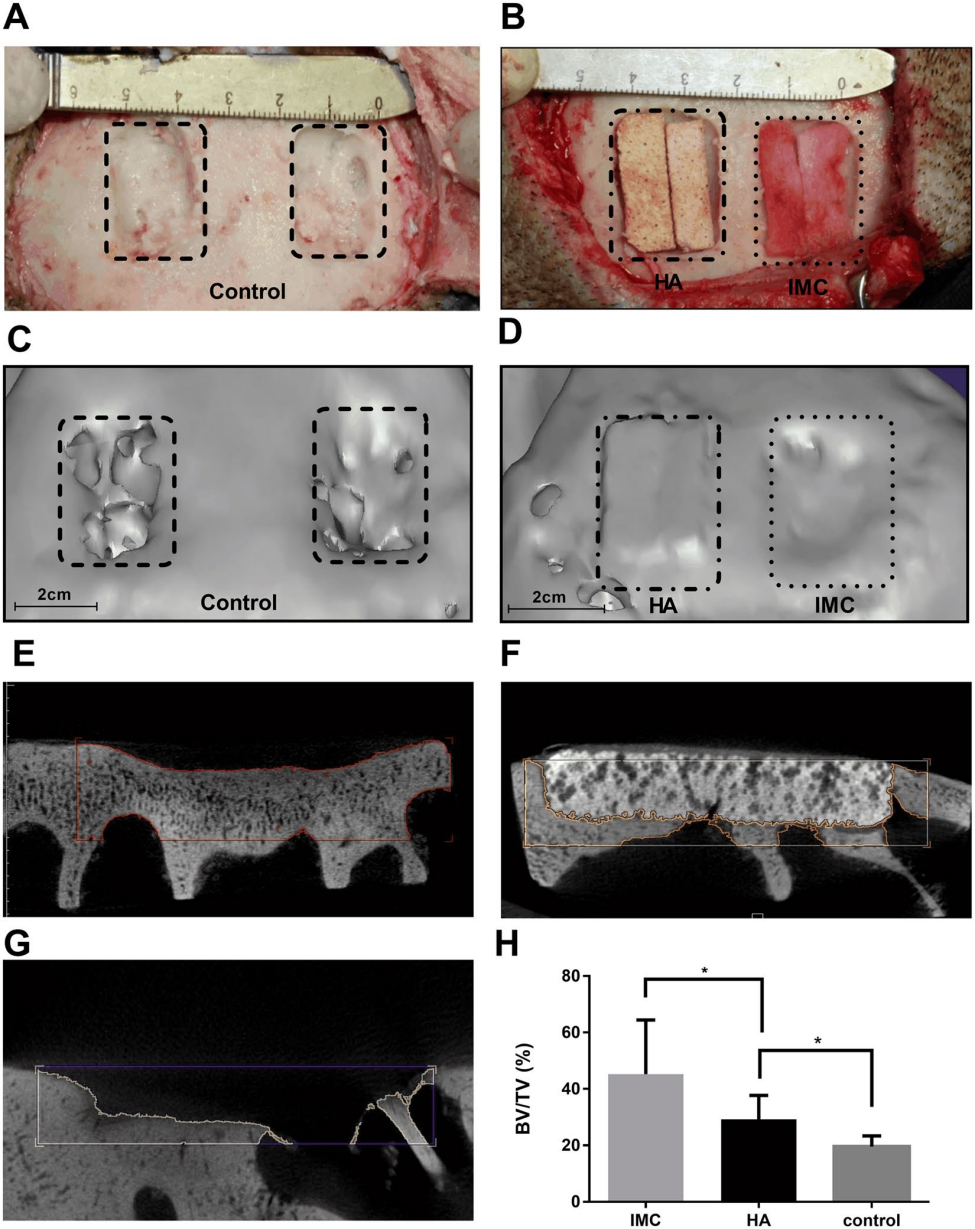
Surgical procedure for producing a non-healing defect and representative CT images of bone regeneration by different scaffolds. (**A**) A non-healing defect of 2 cm width × 3 cm length × 0.5 cm depth in minipig cranium. (**B**) The defects were respectively filled with HA and IMC scaffolds seeded with PDLSCs. (**C**,**D**) Representative 3-D reconstructed images in the control (**C**) and experimental groups (**D**). (**E–G**) Representative cross-section images of defect area after implantation with IMC (**E**) and HA (**F**), and without implants (**G**) for 12 weeks. (**H**) Volume analysis of the defect area based on CT results. Groups labeled with star are significantly different (P < 0.05).

To investigate the bone regeneration potential of the IMC scaffold in large animals, non-healing full thickness defects without periosteum were created in cranial bone of minipigs (Fig. 3A,B). After implantation for 12 weeks, these bone defects were almost filled with fibrous bone structures even at the defect center and the depth of such defects significantly decreased in the IMC group (Fig. 3D,E). By contrast, the defect area in the HA group remained highly radiopaque, and an obvious boundary was observed between the scaffold and defect margin (Fig. 3D,F). The mineral density for the HA group was higher than that for natural bones (Fig. S2), thereby indicating that a large number of HA scaffolds did not degrade in the defect area. For the negative control, only a small amount of neo-bone was formed long the defect margin (Fig. 3C,G). According to the quantitative analysis, the IMC group achieved a significantly higher extent of new bone formation (BV/TV = 45.2 ± 17.7%) than the HA (BV/TV = 29.3 ± 7.7%) and control (BV/TV = 19.6 ± 3.4%) groups (Fig. 3H). Furthermore, the mineral density of the new bones in the IMC group was similar to that of natural bones. (Fig. S2).

### Histological evaluation of regenerated bone in minipigs

**Figure 4:**
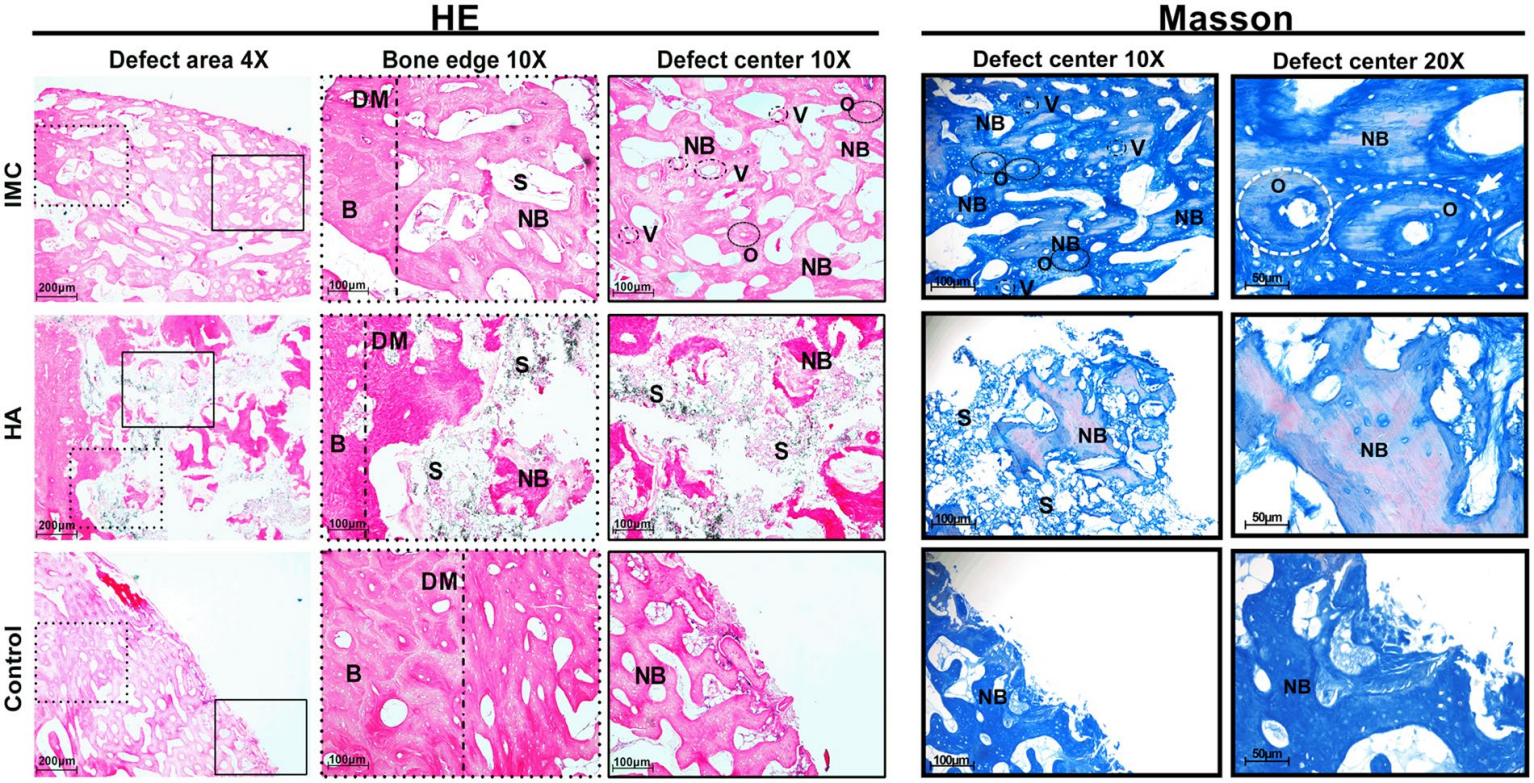
HE and Masson’s trichrome staining of defect area after implantation with different scaffolds for 12 weeks. Plenty neo-bone with osteon and vessles was formed by IMC, whereas limited newly-formed bone and lots of residual scaffolds were observed in the HA group. A small amount of newly-formed bone was seen around the defect margin in the control group. B: natural bone; S: scaffold; NB: new bone; DM: defect margin (black dotted line); O: osteon; V: vessel.

Histological analyses including HE and Masson’s trichrome stainings were performed to observe the microstructure of tissues in the defect area (Fig. 4). In the IMC group, plenty of new bones with few unresorbed scaffold remnants were observed along the defect margin, and the appearance of osteon with osteocytes and blood vessels in the defect center indicating the active formation of neo-bone (Figs 4 and S3). By contrast, limited new bone structure with a large number of undegraded HA remnants was identified in the defect area in the HA group. The residual HA scaffold volume (40.88 ± 6.29%) was six times greater than the residual IMC scaffold volume (6.34 ± 2.14%) (Fig. S4). The remnants of HA scaffolds prevented the establishment of a completely normal architecture of natural bones and blood vessels. In the control group, only a small amount of new bone was seen along the defect area. Consistent with the CT data, the semi-quantitative analysis results (Fig. S3) indicate that the IMC group has a significantly greater amount of new bones than the other groups (P < 0.001).

The Masson’s trichrome staining of the defect center showed that abundant new collagen fibers and a completely normal architecture of natural bone with osteocytes and blood vessels could be observed in the IMC group (Fig. 4). Some mature bones with red dye were also observed in the IMC group. The limited number of new bones with red dye in the HA group showed a relatively high maturity, yet lacked the normal architecture of natural bones. A small amount of new bones with blue dye was observed in the control group.

**Figure 5:**
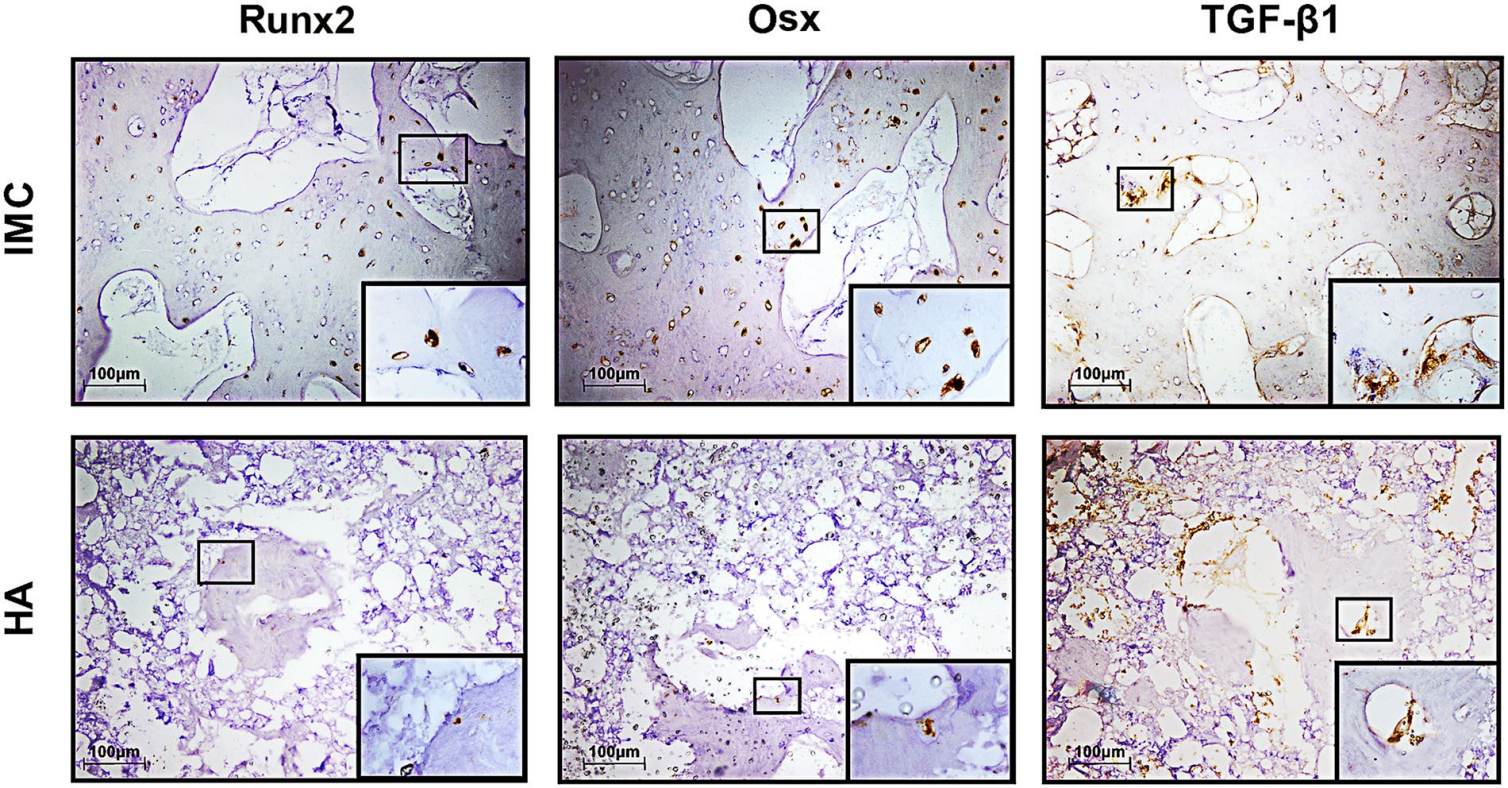
Immunohistochemical staining of Runx2, Osx, and TGF-β1 in the defect areas of the two *in vivo* groups. Runx2, Osx and TGF-β1 were highly expressed in the IMC group, whereas weak or negative staining was observed in the HA group.

Runx2 and Osx are two early transcription factors required for osteoblastogenesis and bone formation^32^. From the immunohistologic staining, Runx2 and Osx were highly expressed in the IMC group, whereas weak or negative staining was observed in the HA group (Fig. 5). These findings indicate that more new bones (Fig. S3) and osteocytes (Fig. S5) are present in the IMC group than in the HA group. The expression level of TGF-β1, which influences growth, differentiation and ECM secretion during bone development^33^ was also higher in the IMC group than in the HA group. The increased expression of TGF-β1 may contribute to the formation of ECM in the IMC group *in vitro*.

### Nanostructure and nanomechanics of newly-formed bone

**Figure 6:**
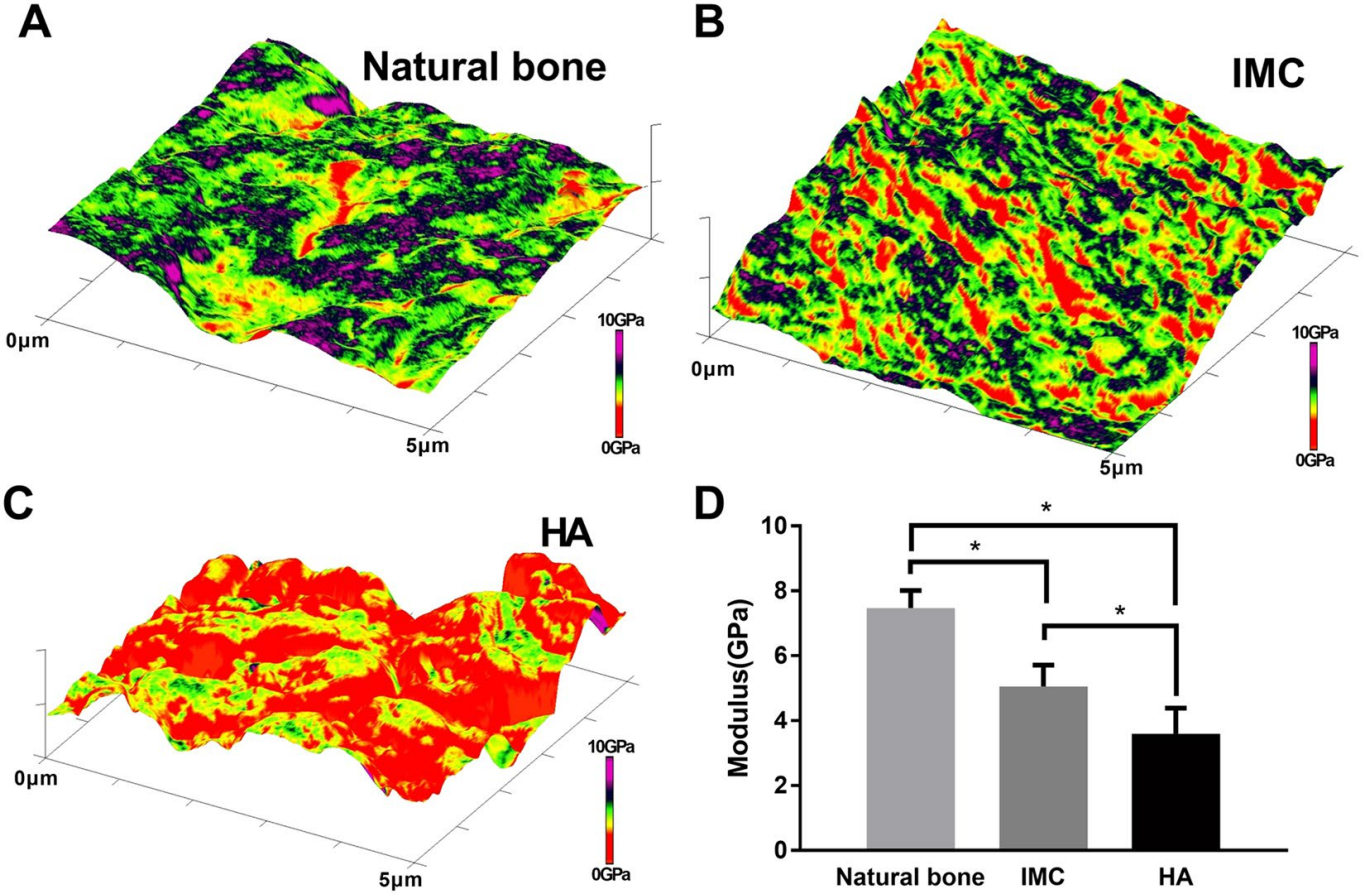
Nanomechanics of bone tissue by AFM. (**A–C**) Representative 3-D AFM property maps of natural bone (**A**), and newly-formed bone in the IMC group (**B**) and HA group (**C**). (**D**) Quantitative analysis of Young’s modulus in different groups. Groups labeled with star are significantly different (*P < 0.05).

The nanostructure and nanomechanics of newly-formed bone in the defect center in the IMC and HA groups were evaluated by AFM using the PeakForce QNM mode (Fig. 6). Mineralized collagen fibrils with distinct cross-banding patterns were identified in the IMC group and were similar to the nanostructure of natural bones. From the 3-D property maps, the modulus distribution of the newly-formed bones in the IMC group was similar to that of natural bones. The quantitative analysis also showed that the new bones formed in the IMC group (5.1 ± 0.7 GPa) possessed improved nanomechanical properties than those formed in the HA group (3.6 ± 0.8 GPa). This might be because that HA induced less neo-bone formation with many voids in the defect center.

## Discussion

The introduction of novel biomaterials for human bone repair requires preclinical safety testing using laboratory animals. With bone anatomy, microstructure, and remodelling process that are similar to those of humans, minipigs have been used as large animal models for the pivotal preclinical testing of human skeletal implants^29–31^. In this study, we first reported the use of biomimetic IMC in combination with autologous PDLSCs for the regeneration of large bone defects in minipigs. Compared with rat critical-sized mandibular defects of approximately 5 mm in diameter in our previous studies^27, 28^, the defect area of approximately 2 cm width × 3 cm length × 0.5 cm depth) in this study is an order of magnitude greater. The IMC still possesses an excellent bone regeneration and vascularization potential. Furthermore, the nanostructure and nanomechanics of the new bone generated in the IMC group were similar to those of natural bones, while the HA promoted disorganized formation of new bones with poor mechanical properties. These measurements by AFM are critical for assessing new bone biofunctions because the bone collagen fibril architecture dramatically affect bone mechanical strength (e.g. osteoporosis)^34^.

Optimum bone grafts have three prerequisites, namely, compatibility with the surrounding tissue, controllable degradation rate, and proper porosity to protect the healing space and allow cell migration and blood vessel ingrowth^35–37^. The cell seeding experiment in this study showed that the porous IMC had an excellent biocompatibility and was suitable for minipig PDLSC attachment and proliferation. To achieve maximum bone generation, we should balance both degradation rate and healing time. After 12 weeks of implantation, the IMC almost degraded and facilitated an abundant new bone ingrowth, while numerous undegraded HA inhibited bone healing and normal bone architecture formation. Although HA, as a main inorganic component of natural bone, seems to be an ideal bone graft, its uncontrollable degradation rate remains a challenge^11, 38^. The HA remnants also increased the volume and mineral density of the regenerated area evaluated by CT, thereby underscoring the importance of histological analysis for assessing new bone formation. Observing the bone regeneration potential of IMC and the degradation rate of HA requires a longer time in our future study. Other criteria for porous scaffolds include porosity and pore interconnectivity to allow cell infiltration and vascularization^39^. The optimal pore size for new bone ingrowth ranges between 100 microns and 400 microns^40^. The IMC has a proper pore size of 148.2 ± 46.5 μm and a more interconnective space than HA, both of which can lead to vascular tissue ingrowth in the IMC group.

Bone regeneration is essentially a process of ECM formation and mineralization^41^. Coaxing appropriate cell-material interactions toward new bone regeneration is a basic premise of biomaterials in tissue engineering. In this study, we demonstrated that the physical and chemical properties of scaffolds could affect the ECM secretion of seeded cells. Limited ECM with calcium nodules was formed on the surface of those cells that were seeded on the inorganic HA scaffold. By contrast, those cells seeded on the IMC composite secreted large amount of membrane-bound matrix vesicles with abundant cell-cell junctions, that resembled the matrix production process of bone formation *in vivo* ^42^. The active ECM secretion *in vitro* may further contribute to bone regeneration *in vivo*. The increased expression of Runx2 and Osx during the bone regeneration process reflects the osteoinductive potential of IMC, which activates more bone-forming cells. TGF-β1 is an important transcription factor for regulating ECM formation and mineralization^43^. The increased expression of TGF-β1 in the IMC group suggests the involvement of the TGF-β1 signaling pathway in the formation of mineralized ECM as induced by IMC.

## Conclusions

This study is the first to use biomimetic IMC in combination with autologous PDLSCs for the regeneration of large bone defects in minipigs. Compared with HA, IMC achieved a significantly higher extent of forming new bones, with the normal architecture of natural bones and blood vessels. The new bones generated in the IMC group also showed the similar nanostructure and nanomechanics to natural bones. Therefore, IMC presents a great potential for treating large bone defects in the future.

## Electronic supplementary material

supporting information
