# Supplementary material for: Bone regeneration in minipigs by intrafibrillarly-mineralized collagen loaded with autologous periodontal ligament stem cells
Source: Sci Rep. 2017 Sep 5;7:10519. doi: 10.1038/s41598-017-11155-7 (PMC5585269; doi:10.1038/s41598-017-11155-7)
Supplement: Supplementary file 1 — supporting information [file 41598_2017_11155_MOESM1_ESM.docx]

Bone regeneration in minipigs by intrafibrillarly-mineralized collagen loaded with autologous periodontal ligament stem cells

Ci Zhang^1^, Boxi Yan^1^, Zhen Cui^2^, Shengjie Cui^1^, Ting Zhang^1^, Xuedong Wang^1^, Dawei Liu^1^, Ruli Yang^1^, Nan Jiang^1^, Yanheng Zhou^1^*, Yan Liu^1^*

^1^Laboratory of Biomimetic Nanomaterials, Department of Orthodontics, Peking University School and Hospital of Stomatology, National Engineering Laboratory for Digital and Material Technology of Stomatology, Beijing Key Laboratory of Digital Stomatology, Beijing 100081, China.

^2^Department of Stomatology, Beijing Tongren Hospital, Capital Medical University, Beijing 100730, China.

* Corresponding author

E-mail: [yanhengzhou@gmail.com](mailto:yanhengzhou@gmail.com) (Y.Z.); orthoyan@bjmu.edu.cn (Y.L.)

**Supplementary Information**

**
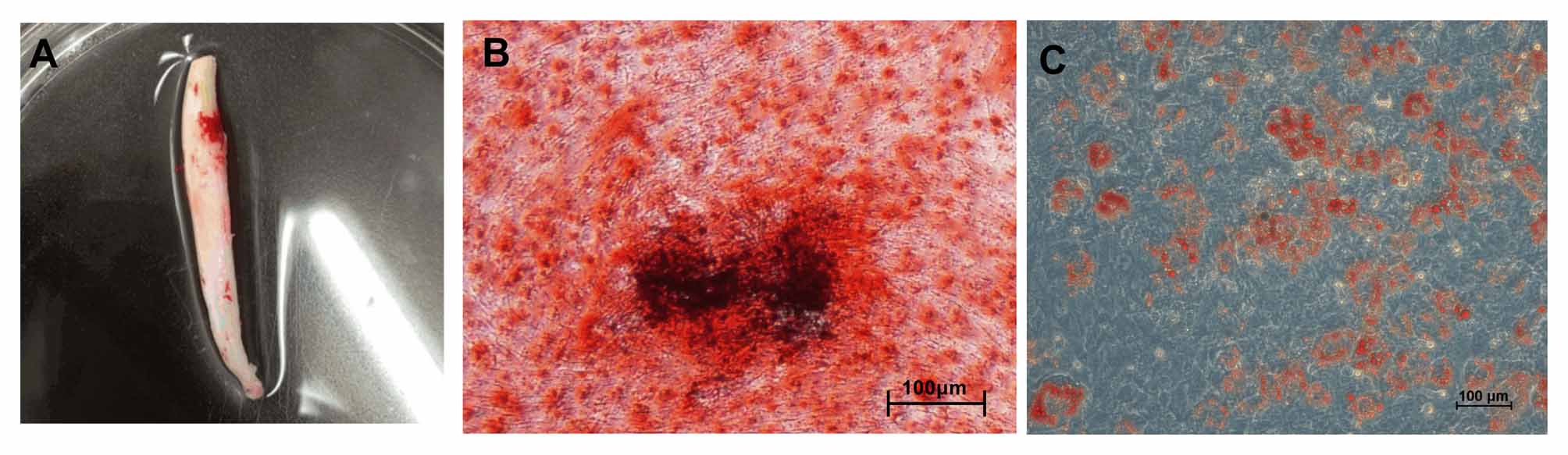
**

**S1 Fig. Multipotent differentiation potential of minipig PDLSCs**

(A) Representative photomicrograph of primary culture of PDLSCs. (B, C) Representative images of alizarin red-S (B) and oil red O (C) stainings. After induction in osteogenic and adipogenic medium for 3 weeks, differentiated PDLSCs showed alizarin red S-positive staining by the formation of mineralized nodules and oil red O-positive staining by the accumulation of lipid clusters respectively.


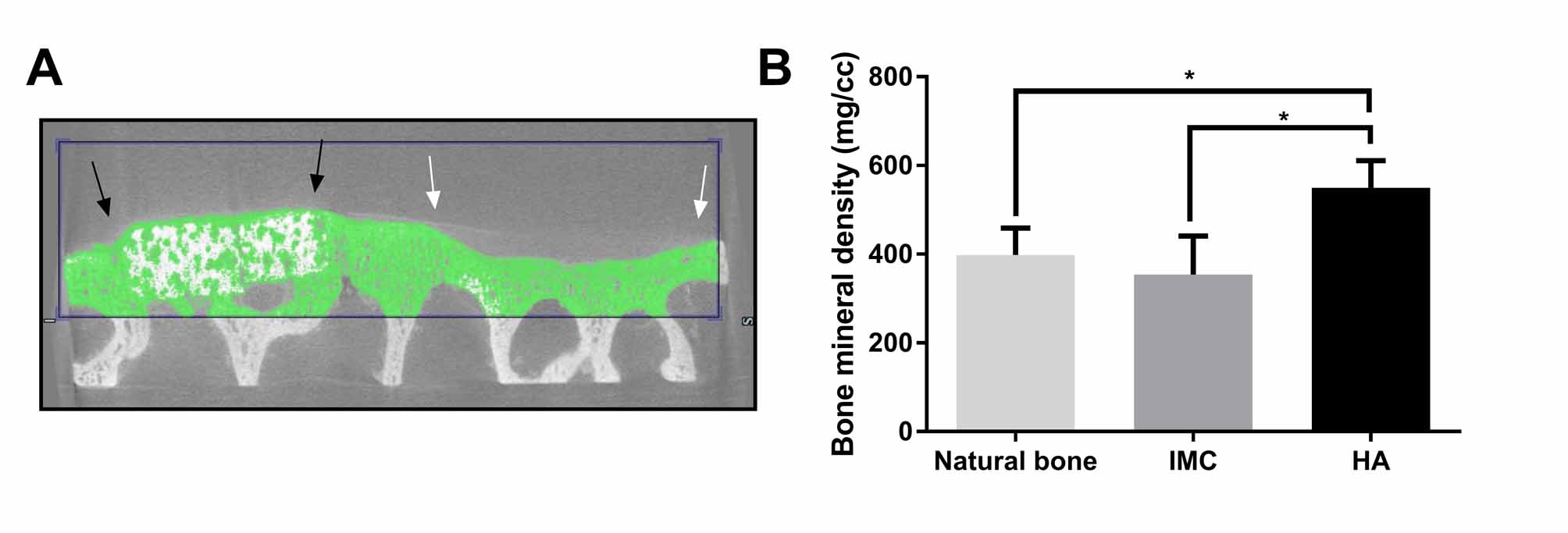


**S2 Fig. Bone mineral density of defect area.**

(A) Cross-section of CT image showing that the mineral density of the neo-bone in the IMC group was similar to that of nature bone (white arrows). A large number of undegraded HA were highly radiopaque (black arrows). (B) Semi-quantitation of bone mineral density (BMD) of natural bone and defect area after implantation with IMC and HA for 12 weeks. Groups labeled with stars are significantly different (*P<0.05).


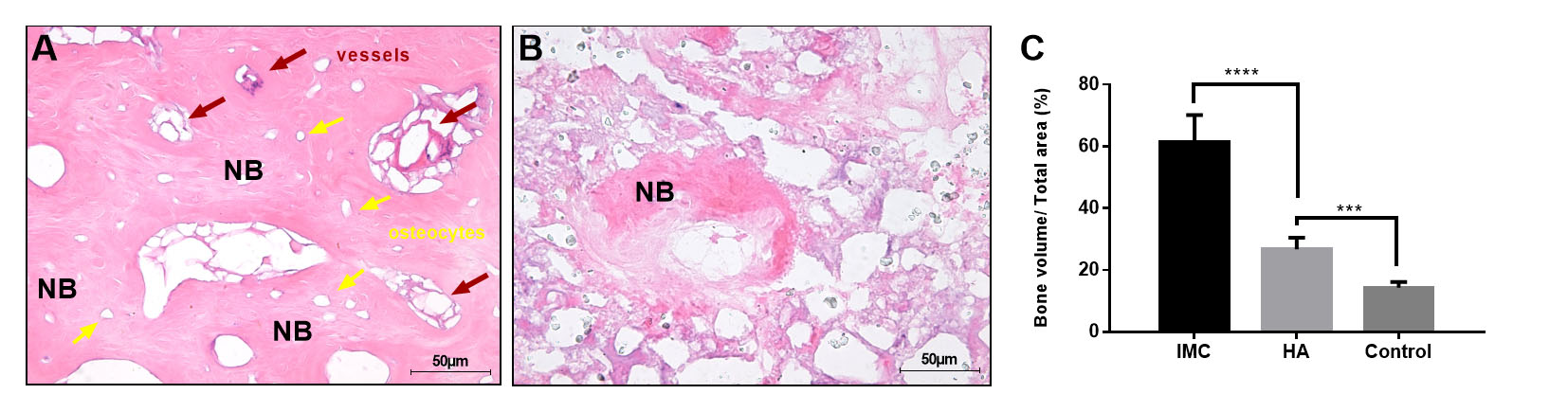


**S3 Fig.** (A, B) High-magnification of HE staining images of the neo-bone formed by IMC (A) and HA (B). NB: neo-bone**.** (C) Semi-quantitative analysis of Bone volume/Tissue volume in each group. Groups labeled with stars are significantly different (***P =0.0001 and ****P<0.0001 respectively).


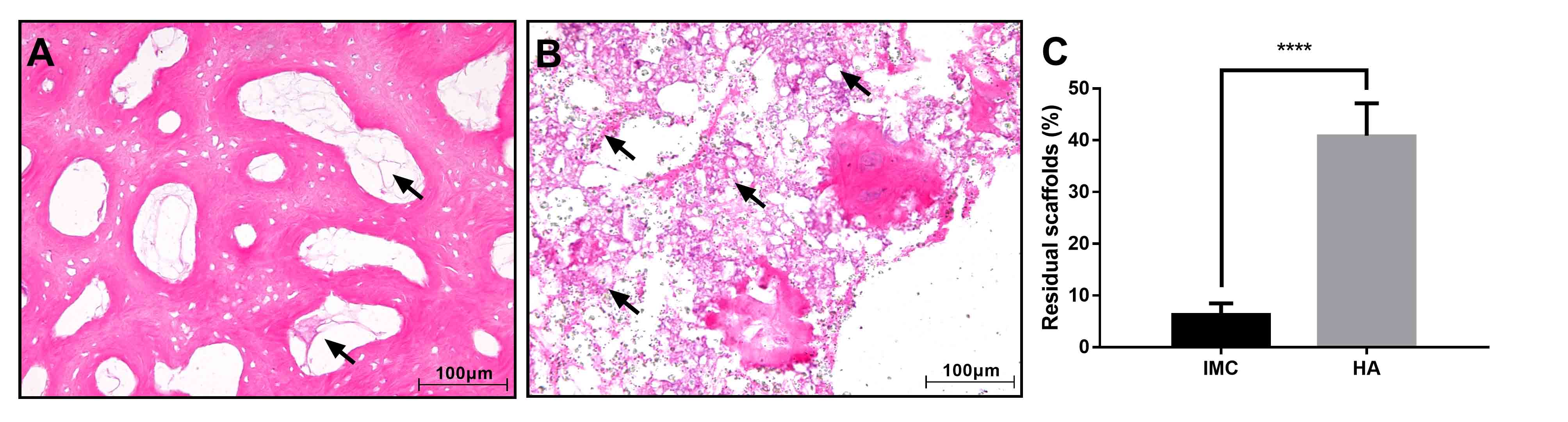


**S4 Fig.** (A,B) Representative HE staining images of defect area after implantation of IMC (A) and HA (B) for 12 weeks. Arrows: residual scaffolds. (C) Semi-quantitative analysis of residual scaffolds in each group. Groups labeled with stars are significantly different (P <0.0001)


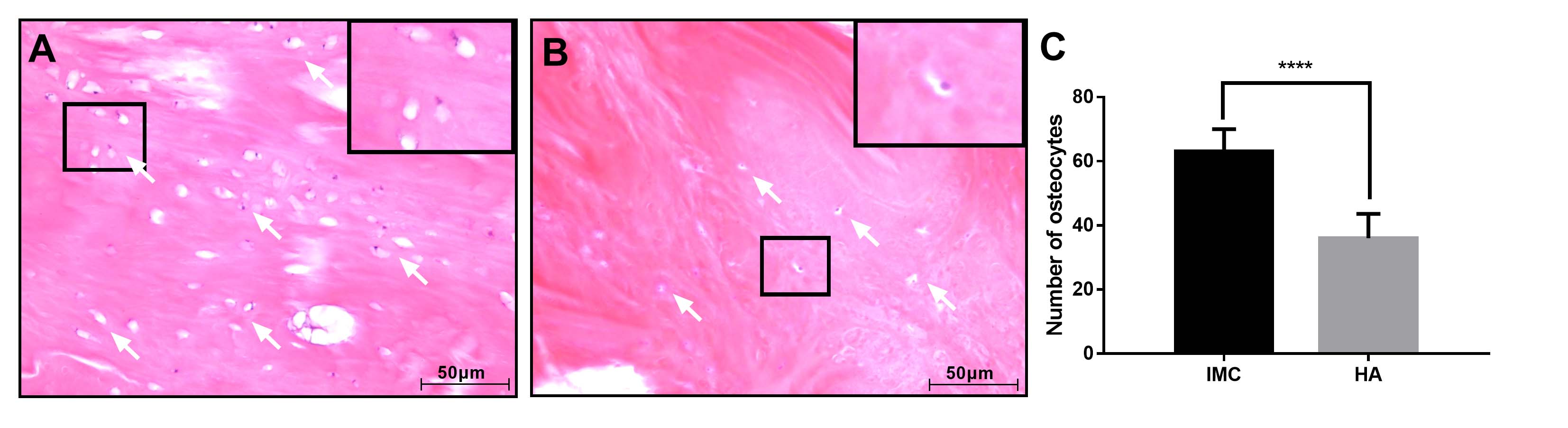


**S5 Fig.** (A, B) Representative HE staining images of the neo-bone formed by IMC (A) and HA (B). (C) Semi-quantitative analysis of the number of osteocytes in each group. Groups labeled with stars are significantly different (P <0.0001)
